# Physicians’ professional identities: a roadmap to understanding “value” in cardiovascular imaging

**DOI:** 10.1186/s12968-016-0274-x

**Published:** 2016-08-26

**Authors:** Eric J. Keller, Robert L. Vogelzang, Benjamin H. Freed, James C. Carr, Jeremy D. Collins

**Affiliations:** 1 Department of Radiology, Northwestern University Feinberg School of Medicine, 737 N. Michigan Ave Suite 1600, Chicago, IL 60611 USA; 2 Department of Medicine-Cardiology, Northwestern University Feinberg School of Medicine, Chicago, IL USA

**Keywords:** Quality improvement, Perceived value, Professional identity, Cardiovascular imaging

## Abstract

**Background:**

Quality improvement efforts in cardiovascular imaging have been challenged by limited adoption of initiatives and policies. In order to better understand this limitation and inform future efforts, the range clinical values related to cardiovascular imaging at a large academic hospital was characterized.

**Materials and methods:**

15 Northwestern Medicine physicians from internal medicine, cardiology, emergency medicine, cardiac/vascular surgery, and radiology were interviewed about their use of cardiovascular imaging and imaging guidelines. Interview transcripts were systemically analyzed according to constructivist grounded theory and combined with 56 previous interviews with interventional radiologists, interventional cardiologists, gynecologists, and vascular surgeons to develop a model describing specialty-specific values. This model was applied to the 15 pilot interviews focused on cardiovascular imaging, highlighting specialty specific differences in values and practice patterns. Transcripts were also reviewed independently by a cardiologist and 2 radiologists followed by a group discussion to assess reproducibility and achieve a consensus regarding the results.

**Results:**

Differences in perceived value of cardiovascular imaging and use of guidelines among physicians were well explained by three value-associated identity categories (managers, diagnosticians, and fixers) that were further differentiated along three axes (broad v. focused-thinkers, complex v. definitive-answer-seekers, and public visibility).

**Conclusions:**

Quality improvement in cardiovascular imaging may be limited by a lack of understanding and incorporation of the complexity of medical culture into ongoing initiatives. Both individually and during policy development, it is important to first understand the complexity of stakeholders’ diverse perceptions of “value,” “quality,” and “appropriateness.”

## Background

Cardiovascular imaging has been identified by the National Quality Forum and Institute of Medicine as an important area for quality improvement, both economically and socially [1, 2]. Past initiatives in this area of healthcare, like others, have yielded some promising results but remain challenged by a lack of sustainability and physician support. For example, a recent meta-analysis [3] found that physician audit, feedback, and education about the American College of Cardiology’s (ACC) Appropriate Use Criteria (AUC) significantly reduced inappropriate use of cardiac imaging. However, other studies that sought to educate and provide feedback about AUC failed to impact behavior, finding that autonomous professionals “don’t want to be told what to do” [4, 5]. Even those studies reporting initial success have found “inappropriate” scan rates to return to pre-intervention rates in as little as one year [6, 7].

When such interventions fail, it can be easy to assume that other physicians do not care or are acting selfishly. Consider Andy Slavitt’s comment regarding the abrupt end of the Center of Medicaid and Medicare Services’ (CMS) Meaningful Use initiative, “we have to get the hearts and minds of physicians back. I think we’ve lost them” [8]. The dominant approach has been to “educate” and/or financially incentivize (through threat or reward) behaviors that groups of experts have defined as appropriate or cost-effective [9]. However, this approach assumes that most physicians share common definitions of appropriateness and value and are primarily driven by economic gains. Since American medicine lacks much of a central authority beyond the government’s involvement in health insurance [10], medical authority is spread across a complex array of professional societies, guidelines, and training experiences, creating and propagating distinct truths and values [11].

Additionally, although physicians value reimbursement, the highest paid physician is not necessarily valued or respected most by his/her colleagues. Physicians are driven by autonomy, mastery, and a sense of purpose, and if a system incentivizes actions that undermine these values, it can “sap” physicians of their internal drive and lead to poor policy adoption [12–14]. Simply put, drivers of professional behavior and perceptions of value are complex [15], and without a better understanding and appreciation of this complexity, even well-intentioned interventions can fail to resonate with those they aim to affect. In other words, it is not a lack of values or interests that limits physicians’ adoption of guidelines and interventions but the range of values and experiences that are present.

Humans’ tendency to form social groups and distinct values is a well described concept in psychology called social identity theory [16]. Past work has illustrated that medical specialties (and any academc discipline) exhibit distinct vocabulary, relics, idols, and thus, cultures that can cause miscommunication and tension [17–20]. However, much of healthcare policy and guideline development has relied on a monocultural view of physicians. This may be due to the fact that the American Medical Association (AMA) and allopathic medicine in general have long emphasized common physician values, objective scientific evidence, and self-regulation [21], leading many to falsely assume that all physicians hold the same values and interpretations of evidence despite perceived personality differences. Few would disagree that an internist is different from a surgeon, but exactly how these professional groups differ in terms of clinical values and decision making remains largely unexplored and underappreciated in healthcare.

Cardiovascular imaging is particularly vulnerable to cultural differences between specialty groups because a significant portion of cardiovascular imaging is performed and interpreted by non-radiologists and the decision to order a particular imaging test may or may not be made by the same speciality responsible for performing and interpreting that imaging. Although surveys and cost-utilization comparisons are useful and easier to collect and analyze, previous anthropologic studies exploring the complexity of clinical decision making and values have required a more sensitive qualitative method called “grounded theory (GT).” GT is a well-validated method for systemically exploring poorly understood social processes through the simultaneous generation and analysis of qualitative data that has been used since the 1960s to understand a wide array of patient and/or physician perceptions [22–26]. However, some applications of GT can be quite time-intensive and laborious, limiting their clinical feasibility. Thus, we used what is called Constructivist GT (C-GT) to facilitate a preliminary investigation of differences in the perceived value of CV imaging between specialties. C-GT expedites traditional GT by not approaching the investigation with a completely blank slate (e.g. knowing we are most interested in perceptions of value) and omitting some analysis steps after main results become apparent [27, 28].

## Methods

### Research strategy

In order to efficiently illustrate value differences, we selected 3 physicians in each of 5 specialties involved in cardiovascular (CV) imaging [internal medicine (IM), cardiology, radiology, emergency medicine (EM), and cardiac/vascular surgery] at a single institution (Northwestern Medicine). Often C-GT requires sample sizes of at least 6–12 to capture the majority of important concepts in each group [29]; however, this pilot investigation sought to merely illustrate the range of values rather than definitively characterize them.

### Semi-structured interview design

Interviews were conducted by a medical student with 2 years experience conducting semi-stractured interviews with physicians (EJK). This approach facilitated a conversational tone and reduced filtered answers due to the interview style and interviewer’s status as a non-threatening member of the medical community [30]. Interviews were introduced as a pilot study to understand how different specialties “use” CV imaging. Specifically, “use” meant any interaction with imaging as part of their professional role, including but not limited to ordering, interpreting, and discussing results with patients. This general word was used to not limit interviewees’ descriptions of their interactions with CV imaging. Physicians were first asked to describe their professional role and use of CV imaging through typical patient interactions. Follow up questions, such as simply “why” or “can you give me an example,” were used to gather specific details and a richer understanding of their clinical reasoning. The interviewer also summarized concepts back to interviewees to confirm understanding, differentiate more vs less important concepts, and establish rapport. Interviewees were then asked more sensitive questions about how their approach compared to others within and outside their field, their use/opinions of CV imaging guidelines, and possible solutions to any expressed concerns with further follow up questions. Finally, physcians were asked what they valued about their specialty and if there was anything else they felt was important that was not discussed.

### Data analysis

All interviews were transcribed verbatim and systematically analyzed according to C-GT [27], using NVivo 10 (QSR International). Key concepts were identified by considering interviewees’ emphases and frequencies of ideas. For example, an initial EM physician interview yielded concepts such as undifferentiated patients, considering worse case scenarios, gatekeeper, efficiency/triage, and imaging to avoid malpractice concerns. Interview concepts from these 15 interviews were compared within and across specialty groups along with 56 other interviews conducted with interventional radiologists, interventional cardiologists, gynecologists, and vascular surgeons about their choice of specialty and approaches to patient care. These additional interviews were conducted in the same style by the same interviewer during the last 2 years. Similar concepts were present in each specialty group but distinct from others. For example, concepts such as undifferentiated patients and gatekeeper were dominant throughout all 3 EM interviews but not present in any of the 68 other physicians interviews we have conducted to date. In light of this, interviews were reassessed to characterize common themes that differentiated physicians’ values and clinical reasoning. Three dominant value-associated identity categories (managers, diagnosticians, and fixers) and three differentiating axes (broad v. focused-thinkers, complex v. definitive-answer-seekers, and public visibility) emerged from comparing and contrasting the values and themes expressed by each specialty. This coding structure was then applied to previous and remaining interviews to test how well it could explain differences in interviewees’ use of CV imaging and guidelines. Transcripts were also reviewed idependently by a cardiologist (BHF) and 2 radiologists (JDC, RLV) followed by a group discussion to assess reproducibility and achieve a consensus regarding the results.

## Results

### Participants

All but 1/15 pilot physician interviewees were men in practice between 3 and 41 years. With the addition of the 56 previous physicians interviews, 15/71 (21 %) were women; the practice experience was 1–41 years, with 16/71 (23 %) private practice physicians and 22/71 (31 %) physicians from practices outside Northwestern in CA, AR, OH, WI, and NC.

Although the identified professional identity groups and values have been grouped below by specialty, the interviews revealed differing degrees of variation within professional groups. In general, smaller, more-focused groups such as interventional radiologists tended to exhibit less variation in their expressed values and opinions than broader groups such as “obstetricians/gynecologists.” Characterizing and comparing the degrees of intra-specialty value variation regarding CV imaging would require additional interviews.

### Value-associated identity categories

“Manager” physicians (e.g. internists and cardiologists) tended to value thinking broadly about many different factors contributing to patients’ symptoms to develop differential diagnoses. Patients’ stories and clinical context were particularly important, which they use to “manage” patients or conditions over relatively long clinical relationships.

“I'm probably like a lot of internists in that we enjoyed the story, hearing the story and trying to use that to make a diagnosis and implement treatment… [We] recognize that you can’t just separate systems, a person’s physical health is connected in many ways across a lot of the systems as well as their psychological and social elements….”

In regards to CV imaging, managers tended to view imaging as one piece of information that needs to be considered in light of and incorporated into the clinical story. Internists described using EKGs and stress tests along with histories and physical exam findings to understand best how to manage patients acutely or chronically. Thus, the value of the imaging was simply part of the larger clinical puzzle, “maybe 10 %.” Although some internists feel comfortable ordering cardiac CTs or MRIs, they preferred cardiologists as both their CV consultants and imagers when patients were high risk or needed advanced imaging:

“…there has been debate on the front lines of internal medicine that when you send a patient for cardiovascular imaging who’s the best person to get that result from. There tends to be a fairly strong consensus that they would prefer a cardiologist to offer their opinion than a radiologist… I have a sense of what a cardiologist may do or the interest they may take and the training they’ve had versus a radiologist who I expect has probably had a lot less clinical experience with someone with heart disease and probably a lot less exposure to cardiovascular investigation in their training.” –Internist

Medical subspecialties such as cardiology shared much of the internist identity: “[As a cardiologist] you are an internist.” Although cardiology is a more focused field, it was defined more in terms of clinical knowledge than anatomy or a technical skill set, e.g., cardiologists described themselves as managing CV diseases/patients rather than fixing hearts. Nevertheless, cardiologists described themselves as valuing procedures and imaging more than other internists, making their use of CV imaging more focused and definitive: they more often described imaging as providing definitive answers and playing a larger role in clinical care. Cardiologists particularly valued advanced imaging such as echocardiography or cinegraphic CMR that let them better appreciate the structure-function relationships that piqued their interest in cardiology as trainees. Their roles as CV imagers varied, but they tended to highly value access to and collaboration with radiologists for imaging interpretation: “My interactions with the radiologists are great. I love their insight… from what I hear it’s mutual.” Nevertheless, it was noted that this is not always the case and can cause tension.

“Diagnostician” identities (e.g. radiology and EM) tended to enjoy “knowing something about everything” and getting to solve diagnostic puzzles during relatively short interactions. However, this makes them particularly dependent on other specialties, and so they often felt that they must continually prove their worth to other physicians: “…this concession on [other clinicans’] part is regarded only as a loan of a talent to be developed by us and then returned, is witnessed by the alacrity with which the [medical specialist] takes over the application of x-ray and radium the moment he can make such a move remunerative.” –Radiologist (1920) [31]

For radiologists, the value of CV imaging was a definitive answer to the clinical problem at hand, quantifying and characterizing diameters, motions, and intensities. They also had a particular appreciation for adjustments that could make modalities faster, safer, or more objective, which they suspected other specialties also valued about them.

EM physicians described themselves as unique in seeing “undifferentiated patients” and thinking in terms of worst case scenarios and efficiency for patients waiting to be seen. Thus, the value of CV imaging was defined in terms of triaging patients. EKGs, CXRs, and sometimes coronary CTAs were valued for efficiently determining if patients should be sent home or admitted to cardiology for further workup. Overall, EM physicians seemed to have a lower threshold for imaging, which they attributed to malpractice concerns as gatekeepers between an organization pushing them to lower costs and patients who expect a 0 % miss rate. They also felt that many general practitioners now rely on them as consultants, “…we’ve become the master clinicians in diagnosis;” yet, like other diagnosticians, EM physicians felt underappreciated by many specialists: “No other specialist will ever tell you this but what they’re really looking for is an emergency medicine consult.”

“Fixer” physicians (e.g. cardiac/vascular surgeons) valued tangible outcomes, “doing” technical tasks, and innovation. “I like to see immediate results. I like to work with my hands.” Because of this, they defined their roles primarily by their procedures and/or anatomic regions: “I like to operate on the heart. It’s what defines cardiac surgery….” Due to this reliance on technical skill and ability, value was primarily defined in terms of technical success of fixing specific problems. For example, CV imaging was valued primarily for determining whether or not a procedure was indicated, planning procedures, and assessing the success of procedures. They tended to trust radiologists to provide more consistent measurements and generate official reports but preferred to use 3D reconstructions and ultrasound/fluoroscopy themselves for surgical planning and intraoperative guidance, respectively.

### Broad v. Focused-thinkers

**Fig. 1:**
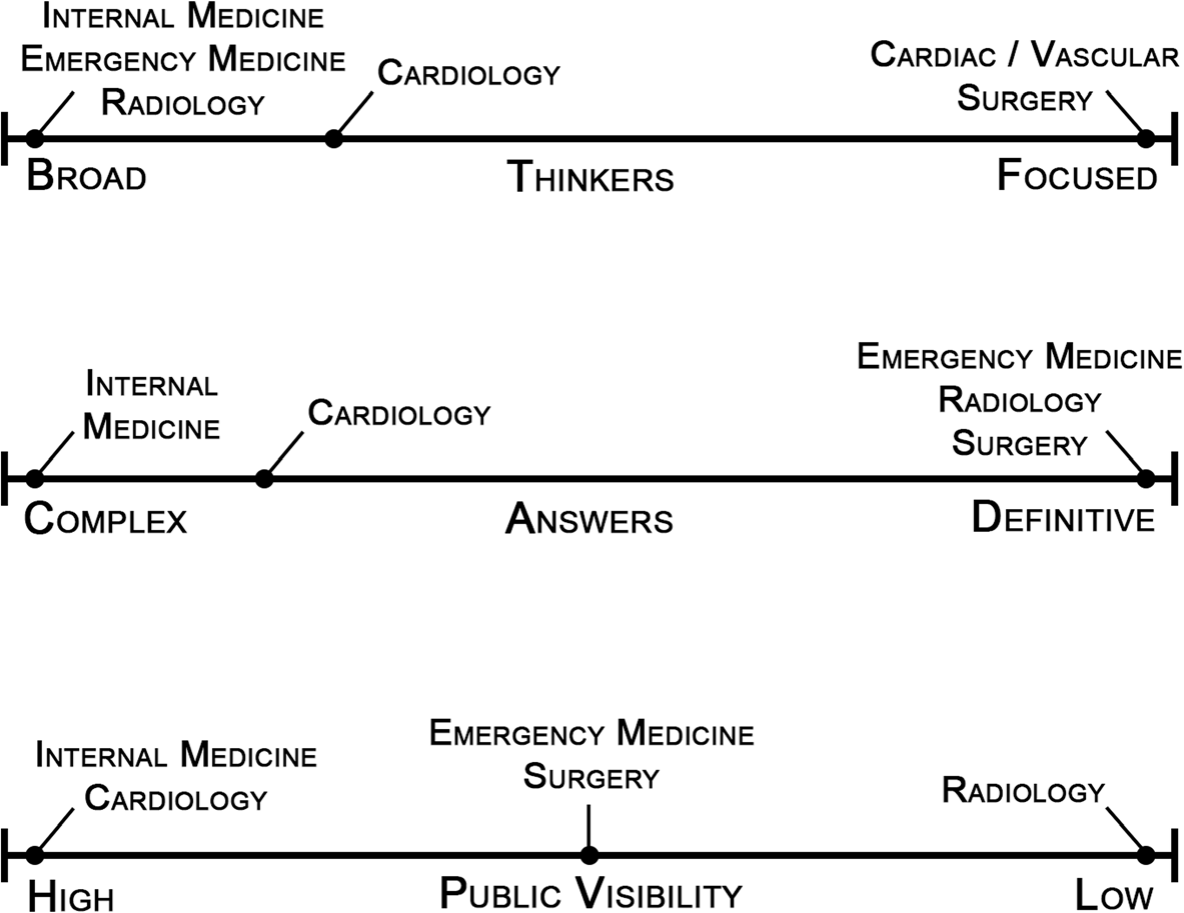
Key axes of physicians’ professional identities and values

Beyond the three categories, physicians’ professional identities were further divided along three key axes, affecting their clinical values and decision making (Fig. 1). Broad-thinkers (e.g. IM, radiology, EM, and cardiologists) valued knowing about many different areas of medicine and casting a broad net whereas focused-thinkers (e.g. surgeons) prioritized narrowing in on a single issue they could control and address. Interestingly breadth of knowledge seemed to garner less respect among colleagues from other specialties than depth. In terms of imaging, focused-thinkers tended to view CV imaging as a tangible means to an ends, whereas broad-thinkers described imaging as an investigation that could lead to a larger number of valuable endpoints.

### Complex v. Definitive-answer-seekers

Physicians that pursue complex answers (e.g. internists, some cardiologists) viewed clinical decision making as more complex and nuanced, often fluid and changing with many caveats and few absolutes. These specialists stressed the importance of clinical context and the interconnections between various aspects of patients’ lives and organ systems. Thus, imaging findings must be accompanied with clinical context to have substantial meaning and rarely offer a definitive conclusion alone.

“I see a person with a heart murmur who also says they’re short of breath. The first question is whether the shortness of breath related to the heart murmur? Or is the heart murmur just incidental to why they have this shortness of breath? …until you understand all the elements of why they have those symptoms and what their cardiac status is you can’t even bring [valve repair] up.” –Cardiologist (2016)

Conversely, definitive-answer-seekers (e.g. radiologists, surgeons, and some cardiologists) tended to draw hard lines while describing their clinical reasoning and use of CV imaging and guidelines. A procedure or test was indicated or not; outcomes were superior or inferior. Not everything was absolute, but too much “gray area” or subjectivity was undesirable. Complex-answer-seekers were far more comfortable with subjectivity and qualitative imaging interpretation whereas surgeons and radiologists prioritized numbers and measurements.

### Public visibility

The final key axis was public visibility, differentiating internists and surgeons from radiologists. In general, this axis paralleled the amount of patient interaction and thus the likelihood of forming clinical relationships with patients. “My radiologist” is rarely used. Interestingly, this axis also seemed to explain a degree of perceived value. For example, there was a clear sense that a cardiologist who can interpret imaging was likely to be valued more by healthcare administration than a radiologist who can counsel patients about cardiovascular disease. Cardiologists’ roles in managing cardiovascular disease has a higher degree of public visibility than radiologists’ roles in interpreting imaging, which attracts wider appreciation for the complexity of this skill. Consider that a radiologist counselling a patient about his/her cardiovascular disease would be met with more skepticism than a cardiologist interpreting a patients’ cardiac MRI. This axis also differentiated EM physicians from radiologists. EM shares the diagnostician need to prove themselves to other physicians, but EM physicians do not have to prove their worth publicly:

“Yeah, one of the funny things about being an ER doc is that if you’re at a cocktail party, everybody thinks that it’s the coolest thing you can do with your life, and if you’re in a room full of other physicians, everybody thinks that you’re a total jackass.” –EM physician (2016)

See Table 1 for summary of identity characteristics and views of CV imaging.

**Table 1:** Summary of specialty differences

Specialty	Identity characteristics	Identity descriptions	Value of CV imaging
Internal Medicine	◊ Manager ◊ Very broad thinker ◊ Complex answers ◊ High public visibility	◊ Patient narrative ◊ Relationship variety ◊ Connecting with patients ◊ Thinking broadly	◊ One piece of the clinical puzzle ◊ “Maybe 10 % of information” ◊ Mainly EKG, stress imaging ◊ Prefer cardiology for further imaging and evaluation
Cardiology	◊ Manager ◊ Broad thinker ◊ Complex/Definitive answers ◊ High public visibility	◊ Structure-function relationships ◊ Procedures/Imaging ◊ Prevention ◊ Managing patients	◊ Diagnosis and management ◊ Prioritize structure-function relationship, e.g., echo, cinegraphic CMR ◊ Collaborative imaging interpretation with radiology
Emergency Medicine	◊ Diagnostician ◊ Broad thinker ◊ Definitive answers ◊ Moderate public visibility	◊ Efficiency ◊ Undifferentiated patients ◊ Gatekeepers ◊ Variety of patients	◊ Triage patients / Rule out worst case scenario ◊ Mainly EKG, CXR, echo, maybe coronary CT ◊ Refer to cardiology for further testing
Radiology	◊ Diagnostician ◊ Broad thinker ◊ Definitive answers ◊ Low public visibility	◊ Technology ◊ Innovation ◊ Knowing something about everything ◊ Consultant	◊ Provide objective answer to clinical question and make patient better ◊ Minimal “gray area” ◊ Right imaging for the right patient ◊ Collaborative imaging interpretation with cardiology
Vascular/Cardiac Surgery	◊ Fixer ◊ Focused thinker ◊ Definitive answers ◊ Moderate public visibility	◊ Working with hands ◊ Technical work with immediate outcome ◊ Ability to *do/fix* something	◊ To determine appropriateness of surgery, surgical planning, surgical follow up ◊ Prioritize 3D CTA or intraoperative ultrasound/fluoroscopy ◊ Radiology does official report but we also interpret imaging

### Use of imaging guidelines

Identity differences also seemed to predict perceptions of imaging guidelines as well as the content and syntax of guidelines themselves. Radiologists tended to view ACR’s appropriateness criteria positively and felt their use of imaging was used to create those guidelines rather than the other way around. However, EM physicians and internists found these criteria less helpful for triaging patients and guiding management, respectively. They instead preferred guidelines from the ACP or ACC and imaging reports from cardiologists. When asked why, both groups mentioned that their time was limited and they did not get a clear sense of how to incorporate the criteria into their clinical thinking. It was not so much that they viewed other specialty guidelines as incorrect but that these guidelines were not as relatable to their professional roles and goals. Cardiologists and surgeons felt they were familiar with ACR’s criteria but cardiologists said they would prioritize the ACC if recommendations differed and surgeons felt these criteria often did not apply to their use of imaging. Again, these perceptions appear to be a reflection of relatability and trust of one’s group.

Even in comparing guidelines themselves one can find the same professional value differences. For example, ACP guidelines tend to emphasize the importance of the clinical history and physicial prior to tests which build upon the clinical story and narrow differentials. Conversely, histories and physical exam findings are rarely mentioned in ACR guidelines that instead focus on choosing the “best test” for specific questions. Thus, it is not surprising that internists who seek complex answers would not find ACR guidelines as relatable, and even cardiologists who find these guidelines more relatable would prioritize ACC guidelines as the product of their mentors, past co-residents/fellows, and evaluators. What obscured the perception of these specialty value differences was the idea of “objective” medical evidence. Clinicians across specialties seemed to assume their trusted organization had correctly evaluated and presented all available evidence, implying differing guidelines must have overlooked or misinterpreted evidence.

## Discussion

Understanding physicians’ distinct professional identities and values can not only help physicians individually but can facilitate policy/guideline development that better resonates with diverse senses of value, fostering wider adoption and support. Our 71 physician interviews over the last two years have revealed remarkably consistent language, values, and opinions within each specialty while differing considerably from those of others. This led us to develop a model to describe these differences which we applied to our 15 most recent pilot interviews, focusing on the perceived value of CV imaging across 5 specialties. This model requires futher refinement and validation in a larger, multicenter study, but our preliminary data suggests this approach may serve as a powerful roadmap to collect data for policy development in CV imaging as well as other areas of imaging.

On an individual level, practicing physicians often encounter cultural differences that can quickly be perceived only as selfishly motivated turf wars [32, 33]. Common examples include fixers feeling that managers waste time excessively forming differentials or recommending less effective interventions and managers criticizing fixers for neglecting important clinical context, treating patients as events, and over-prioritizing their own procedures to maximize profits. It can be easy to conclude that we should try to abolish such differences, but dividing into social groups is a natural human tendency [16] and competition can be a positive source of innovation and work ethic. As put by one radiologist, “friction creates energy… We need people to think differently. We need people to challenge others.” Better collaboration exists not where there are less identity differences but where individuals are more aware of such differences and better respond to them. For example, a radiologist may need to adjust his/her report not only for the clinical question at hand but the specialist asking the question.

**Fig. 2:**
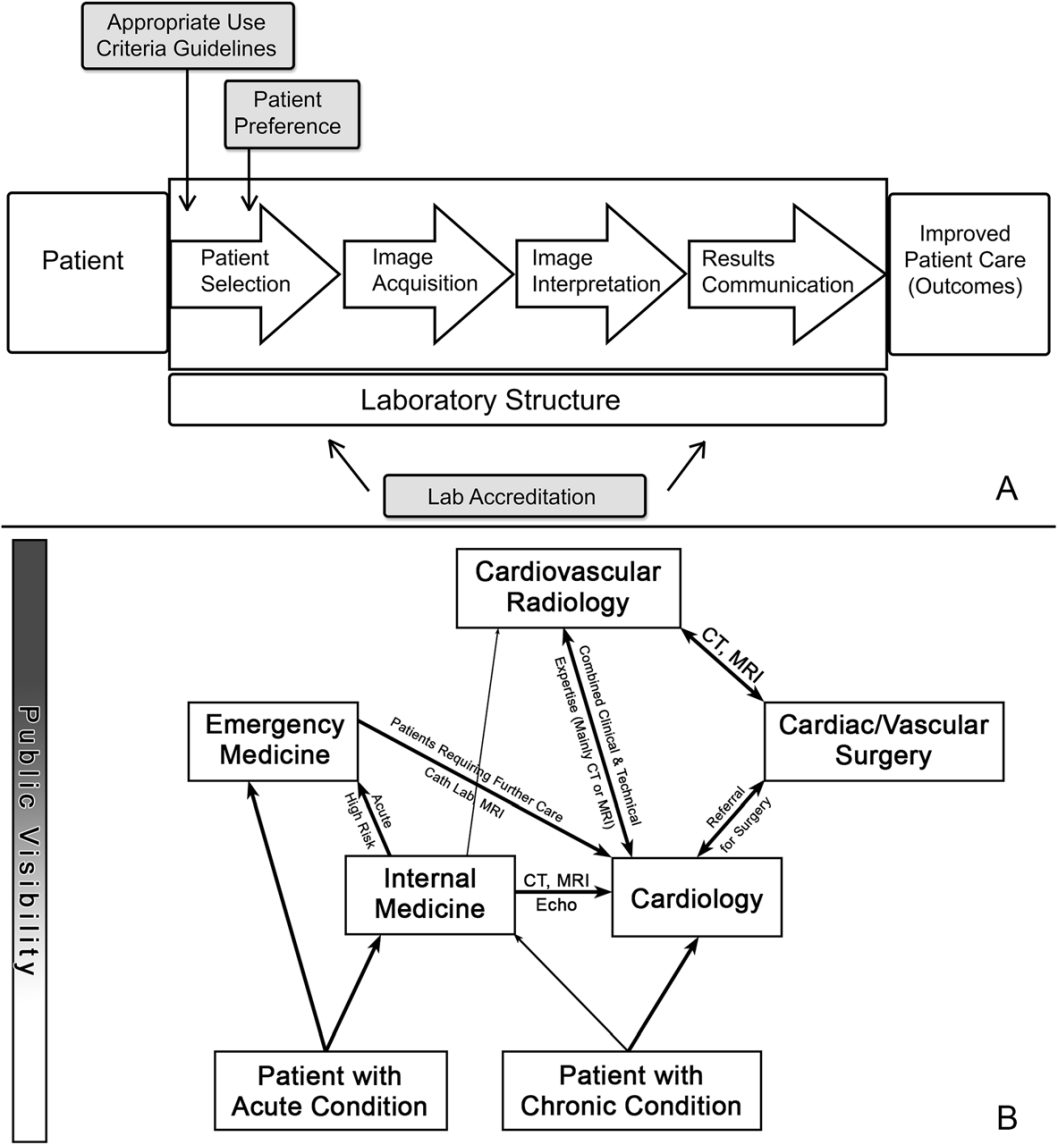
Dimensions of care framework for evaluating quality of cardiovascular imaging (**a**) proposed previous. Reprinted with permission from the Methodist DeBakey Cardiovascular Journal. Patient selection referral pattern for CV imaging among 15 physicians at Northwestern Memorial Hospital (**b**)

In terms of policy development, casting a broad net and working inductively from a richer understanding of stakeholders’ unique and common values seems critical for complex concepts such as “quality” and “appropriateness.” Our results suggest that previous descriptions of “major domains” for imaging quality improvement [34] may fail to appreciate the complexity of medical culture. Common descriptions involve a flow of care events from patient selection (using patient preferences and appropriateness criteria) to results communication (Fig. 2a). Our interviews yielded a similar flow but a complex referral pattern for patient selection (Fig. 2b). This suggests specialty-specific initiatives may be necessary to affect behavior. For example, having cardiologists speak to internists about imaging practices is likely to be more effective than sending radiologists to ‘educate’ them. Quality CV imaing for EM may require addressing malpractice concerns and look different than quality imaging in a cardiology clinic. Additionally, descriptions of the “right” approach to patient selection and results communication have seemed largely physician-centric and specialty-specific. Thus, there is considerable need to better understand how different specialists *and patients* define value and quality related to these two key steps of CV imaging. In other words, there is a need in healthcare for better specialty role definition and a collective understanding that the right course of action for diagnostician can and probably should differ from that of a manager without either being wrong.

Finally, it is worth noting that our identity categories and axes were not only internally valid, but seem to have persisted since each specialty’s formation. After advancements in anesthesia and antisepsis in the late 19th century, surgical techniques expanded rapidly and there became a clear difference between seasoned and amateur surgeons. Thus, the American College of Surgeons was founded in the early 20th century and worked to limit hospital privileges to only a small group of surgeons they certified, which served as a model for other specialty boards [10, 35]. Because of their emphasis on technical skills and abilities, boundaries could be more sharply drawn, making fixers particularly prone to inter-specialty competition. As new technologies such as imaging became available, their value centered around how it allowed fixers to better perform the procedures and problem solving that defined them.

Conversely, there was not a clear initial need for specialization of managers as groups defined more by there breadth of clinical knowledge [10]. Since a cardiologists’ clinical knowledge is built upon his/her internist identity, it is difficult to separate the two. This is likely why manager groups have less clearly defined borders and are content being subspecialties. Thus, the value of imaging and reports has relied on its integration into and support of clinical understanding and management.

Finally, diagnosticians evolved in parallel with multidisciplinary hospitals and advances in medical technology and remain closely tied to the developments that gave them birth. Soon after the discovery of X-rays in 1895, roentgenologists sought to be recognized by other clinicians not only as someone to facilitate use of this new technology but also provide an interpretation or treatment as clinicians. Ever since, radiologists’ values have remained rooted in using technology to provide unparalleled clinical information that could not have obtained without their interpretation or guidance:

“…findings should be interpreted always in conjunction with the laboratory and clinical findings… [A Radiologist is] not a marker of pretty pictures.” -Radiologist (1912) [36]

“…a real radiologist has to be more than a photographer. He should be a very versatile general practitioner specializing in radiology… A great part of the general medical profession, however, does not realize this and some physicians feel that a six weeks’ course will make them fairly familiar with x-ray work.” –Radiologist (1930) [37]

“The ultimate objective is to find out what’s wrong with a patient… it is not just a simple report as you say, it’s deciding what is the most appropriate way of making that diagnosis …[radiologists] have to be familiar with the full spectrum of disease…” –Radiologist (2016)

## Conclusions

If we are to develop imaging guidelines and practices that resonate with providers’ and patients’ diverse senses of value and quality, it seems necessary to first understand the range of perceptions in stakeholders’ own words. Such an understanding requires a qualitative method sensitive enough to capture this complexity, providing a critical roadmap for future healthcare quality initiatives.

## Abbreviations

ACC: American college of cardiology
ACP: American college of physicians
ACR: American college of radiology
AMA: American medical association
AUC: Appropriate use criteria
C-GT: Constructivist grounded theory
CMS: Center of medicaid and medicare services
CV: Cardiovascular
EM: Emergency medicine
